# MHC-II Signature Correlates With Anti-Tumor Immunity and Predicts anti-PD-L1 Response of Bladder Cancer

**DOI:** 10.3389/fcell.2022.757137

**Published:** 2022-02-11

**Authors:** Ruibin Yi, Shuo Hong, Yueming Zhang, Anqi Lin, Haoxuan Ying, Weidong Zou, Qiongyao Wang, Ting Wei, Quan Cheng, Weiliang Zhu, Peng Luo, Jian Zhang

**Affiliations:** ^1^ Department of Oncology, Zhujiang Hospital, Southern Medical University, Guangzhou, China; ^2^ Department of Neurosurgery, Xiangya Hospital, Center South University, Changsha, China

**Keywords:** MHC-II signature, immune checkpoint inhibitor, immune response, prognosis, bladder cancer

## Abstract

A large proportion of anti-tumor immunity research is focused on major histocompatibility complex class I (MHC-I) molecules and CD8^+^ T cells. Despite mounting evidence has shown that CD4^+^ T cells play a major role in anti-tumor immunity, the role of the MHC-II molecules in tumor immunotherapy has not been thoroughly researched and reported. In this study, we defined a MHC-II signature for the first time by calculating the enrichment score of MHC-II protein binding pathway with a single sample gene set enrichment analysis (ssGSEA) algorithm. To evaluate and validate the predictive value of the MHC class II (MHC-II) signature, we collected the transcriptome, mutation data and matched clinical data of bladder cancer patients from IMvigor210, The Cancer Genome Atlas (TCGA) databases and Gene Expression Omnibus (GEO) databases. Comprehensive analyses of immunome, transcriptome, metabolome, genome and drugome were performed in order to determine the association of MHC-II signature and tumor immunotherapy. We identified that MHC-II signature is an independent and favorable predictor of immune response and the prognosis of bladder cancer treated with immune checkpoint inhibitors (ICIs), one that may be superior to tumor mutation burden. MHC-II signature was significantly associated with increased immune cell infiltration and levels of immune-related gene expression signatures. Additionally, transcriptomic analysis showed immune activation in the high-MHC-II signature subgroup, whereas it showed fatty acid metabolism and glucuronidation in the low-MHC-II signature subgroup. Moreover, exploration of corresponding genomic profiles highlighted the significance of tumor protein p53 (TP53) and fibroblast growth factor receptor 3 (FGFR3) alterations. Our results also allowed for the identification of candidate compounds for combined immunotherapy treatment that may be beneficial for patients with bladder cancer and a high MHC-II signature. In conclusion, this study provides a new perspective on MHC-II signature, as an independent and favorable predictor of immune response and prognosis of bladder cancer treated with ICIs.

## Introduction

Immune checkpoint inhibitors (ICIs) have revolutionized bladder cancer (BC) treatment options (Antoni et al., 2017). Urothelial carcinoma accounts for approximately 80–90% of bladder cancers, with the remainder consisting of adenocarcinoma, squamous cell carcinoma, and other rare variant histologies (Willis and Kamat, 2015). Currently, ICIs are used as the first-line treatment for BC patients who are PD-L1 positive and cisplatin-ineligible (Sanli et al., 2017). However, beneficial and long-lasting responses to ICIs only occur in 20–30% of BC patients (Nadal and Bellmunt, 2019). Reliable and promising biomarkers to assess the therapeutic sensitivity toward ICIs are urgently needed. Previous studies have explored biomarkers such as tumor mutation burden (TMB), PD-L1 expression level and fibroblast growth factor receptor 3 (FGFR3) alterations (Rosenberg et al., 2016). Additionally, a series of microRNAs (including miR-608 (Liang et al., 2017), miR-193a-3p (Deng et al., 2014), and miR-138-5p (Yang et al., 2016)) have been demonstrated serve promising biomarkers for the prognosis and drug treatment response in BC. (Deng et al., 2014; Yang et al., 2016; Liang et al., 2017) However, these single biomarkers have several limitations including low accuracy and specificity, insufficiently verified cutoff value, and low frequency incidence (Marincola et al., 2000; Wang et al., 2018). A recent study in BC patients found that tumor response to PD-L1 blockade could be enhanced by blocking transforming growth factor β (TGFβ) with stable and reliable results (Mariathasan et al., 2018), suggesting that the use of gene signatures over single biomarkers may be more accurate in predicting the efficacy of immunotherapy.

As research has historically considered cytotoxic CD8^+^ T cells to play a primary role in immune-mediated tumor killing, efforts to understand how immunotherapy elicits anti-tumor immunity have focused mainly on CD8^+^ T cells and MHC-I molecules (Goodman et al., 2020; Shklovskaya et al., 2020). However, there is mounting evidence in support of cytotoxic CD4^+^ T cells as potential anti-tumor agents, involved in MHC-II-mediated tumor killing (Nagasaki et al., 2020; Oh et al., 2020; Sacher et al., 2020). This indicates a need to redefine the current understanding of the role of these elements in anti-tumor immunity. Additionally, studies have demonstrated that MHC-II neoantigens have key functions in anti-tumor immunity which do not overlap with MHC-I neoantigens (Alspach et al., 2019), something which is important to consider when determining which patients will benefit most from immunotherapy. Evidence exists in the literature which shows a connection between high MHC-II expression and prolonged progression-free survival (PFS) in non-small cell lung cancer (NSCLC) patients after immunotherapy (Yang et al., 2020). Studies also reported the capacity of tumor MHC-II in predicting immunotherapy response in breast cancer (Gonzalez-Ericsson et al., 2021) and melanoma (Johnson et al., 2016; Rodig et al., 2018). Meanwhile, previous studies have found that elevated expression of tumoral MHC Class II positively correlates with BCG tumor immunotherapy in BC (Ikeda et al., 2002; Antonelli et al., 2020). However, the role of MHC-II in tumor immunotherapy of patients with BC has not been thoroughly researched and reported.

Here, we defined a MHC-II signature for the first time by calculating the enrichment score of MHC-II protein binding pathway from the Molecular Signatures Database (MSigDB) with a single sample gene set enrichment analysis (ssGSEA) algorithm. Based on analysis of an ICI-treated bladder cancer cohort (ICI-cohort), we found that the MHC-II signature serves as an independent and robust biomarker for predicting favorable immune response and prognosis in BC patients who received immunotherapy. This was also verified by analyzing Gene Expression Omnibus (GEO) cohorts treated with immunotherapy. Additionally, comprehensive analyses of immunome, transcriptome, metabolome, genome and drugome were performed to characterize the association of MHC-II signature and tumor immunotherapy. These results were verified by The Cancer Genome Atlas Bladder Cancer (TCGA-BLCA) cohort.

## Materials and Methods

### Data Collection and Preprocessing

To evaluate the relationship between the MHC-II signature and the prognosis of bladder cancer patients treated with ICIs, we collected data from an ICI-cohort (*n* = 348) (Samstein et al., 2019). The genome, transcriptome, and related clinical data of bladder cancer patients treated with anti-PD-L1 drugs (atezolizumab) in this cohort were downloaded from: http://research-pub.gene.com/IMvigor210CoreBiologies (Mariathasan et al., 2018). The same data types from the TCGA-BLCA cohort (*n* = 350) were downloaded using the TCGAbiolinks R package (Colaprico et al., 2016). To verify the predictive ability of the MHC-II signature on the efficacy of immunotherapy, we collected the transcription and survival data of two additional cohorts treated with immunotherapy from the GEO. These cohorts contained the following: GSE19423, patients with bladder cancer (*n* = 48) treated with BCG immunotherapy (Kim et al., 2010); GSE176307, patients with bladder cancer (*n* = 87) treated with anti-PD-L1 drugs (Rose et al., 2021). RNA-seq count data were converted into Transcripts Per Million (TPM) (Wagner et al., 2012) in order to calculate gene signature scores.

### Inference of MHC-II Signature Level

To infer the quantity of MHC-II signature, we first obtained the gene set related to the MHC-II protein binding pathway from the MSigDB (Liberzon et al., 2015) (Supplementary Table S1). We then used the gene set variation analysis (GSVA) algorithm (Hänzelmann et al., 2013) and the following gene sets to estimate pathway enrichment scores (ESs) for each sample: Gene Ontology (GO) (The Gene Ontology Resourc, 2019), Kyoto Encyclopedia of Genes and Genomes (KEGG) (Kanehisa et al., 2016), REACTOME (Croft et al., 2011) and HALLMARK (Liberzon et al., 2015). In this way, the ES score serves as a reflection of the quantity of MHC-II gene signature. Kaplan–Meier (KM) analyses were performed on the ICI-cohort and GEO cohorts based on their MHC-II signature scores. The cutoff values dividing the patients into high MHC-II signature score (MHC-H) and low MHC-II signature score (MHC-L) groups were determined based on the association between survival outcome and MHC-II signature scores. This was analyzed in each dataset separately using the survminer R package. Due to the lack of treatment information for the TCGA-BLCA cohort, we divided the patients into MHC-H and MHC-L groups according to the median level of the MHC-II signature score of all samples.

### Immune Characteristic Analysis

To explore the association between MHC-II signature and anti-tumor immunity, we integrated several algorithms to estimate immune infiltration in the ICI-cohort and TCGA-BLCA cohort. First, we used the MCP-counter (Becht et al., 2016) and EPIC (Racle and Gfeller, 2020) to compare the infiltration of immune cells in the MHC-H group and the MHC-L group, respectively. Then, xCell (Aran et al., 2017) algorithms was employed to further compare the specific subtypes of immune cells with infiltration differences between the two groups. All differential gene expression (DGE) analyses were conducted using the DESeq2 R package (Love et al., 2014). DEGs between the MHC-H and MHC-L groups with adjusted P < 0.05 were limited for subsequent analyses. The adjustment P value was calculated using the Benjamini-Hochberg correction (Hochberg and Benjamini, 1990). We then compared the expression difference of the MHC-H and MHC-L groups between immune cell-related genes reported by Charoentong et al. (2017) and immune-related genes reported by Thorsson et al. (2018), respectively.

### Functional and Pathway Enrichment Analyses

The DEGs generated by the DESeq2 package were used as the input, and the genes were sorted according to their logFC values. A gene annotation enrichment analysis was performed using the clusterProfiler R package (Yu et al., 2012) to identify GO (The Gene Ontology Resourc, 2019) as well as the KEGG (Kanehisa et al., 2016) terms; this analysis used a strict cutoff of P values less than 0.05 and a false discovery rate (FDR) lower than 0.05. We also performed gene set enrichment analysis (GSEA) (Subramanian et al., 2005) on the adjusted expression data for all transcripts. Only adjusted values where P < 0.05 were considered to be significant. GO, KEGG and Reactome gene sets came from the MSigDB (Liberzon et al., 2015).

### Mutation Characteristics

To identify the mutation characteristics related to the MHC-II signature, we analyzed the mutation and clinical data of the ICI-cohort and the TCGA-BLCA cohort. A small portion of patients who lacked relevant clinical information were excluded. The ComplexHeatmap R package (Gu et al., 2016) was used to visualize the mutation and clinical landscape of the ICI-cohort and the TCGA-BLCA cohort. Maftools R package (Mayakonda et al., 2018) was used to visualize the mutation sites of high-frequency mutant genes and to explore the co-mutation and mutual exclusion between them. The mutation data of the bladder cancer samples reported by Samstein et al. were obtained by targeted next-generation sequencing (NGS; MSK-IMPACT). The mutation data of the TCGA-BLCA cohort were downloaded using the “TCGAbiolinks” R package (Colaprico et al., 2016). The TMB and neoantigen load (NAL) data for the TCGA-BLCA cohort were obtained from a published study (Thorsson et al., 2018).

### Predictive Value of the MHC-II Signature for Drug Sensitivity

Based on the Genomics of Drug Sensitivity in Cancer (GDSC) database (Yang et al., 2013), we used the “pRRophetic” R package (Geeleher et al., 2014) to estimate the IC50 value of each bladder cancer sample in the ICI-cohort and the TCGA-BLCA cohort for 138 drugs. Next, the drugs that showed significant difference in sensitivity toward the MHC-H group versus the MHC-L group were obtained for further analysis (P < 0.05). To ensure the difference in drug sensitivity was related to the MHC-II signature, the Pearson correlation analysis was performed for each of these drugs based on their IC50 values and MHC-II signature scores. Only P values less than 0.05 were considered to be significant. Based on this analysis in addition to previous literature, the drugs with significant differences in drug sensitivity between the two groups were identified (ICI-cohort, 35 kinds; TCGA-BLCA cohort, 40 kinds). We also performed the mechanism of action (MoA) analysis on the identified drugs to discover other candidate compounds that may have benefit combined with immunotherapy for bladder cancer patients with high MHC-II signature (Subramanian et al., 2017).

### Statistical Analysis

Cox proportional hazards models were used to perform univariate and multivariate analyses. The Kaplan-Meier method was used to generate survival curves for the subgroups in each data set, and the log-rank test was used to determine statistically significant differences. The Shapiro-Wilk normality test was used to test the normality of variables. The Mann–Whitney U test was used to estimate the immune cell related gene expression profile and immune related gene expression profile for the MHC-H and MHC-L groups and were compared to TMB, tumor neoantigen burden (TNB), and immune cell abundance results. All statistical analyses were completed in R (version 4.0.2).

## Results

### High MHC-II Signature Predicts the Favorable Prognosis and Immune Response of Immune Checkpoint Inhibitors

**FIGURE 1:**
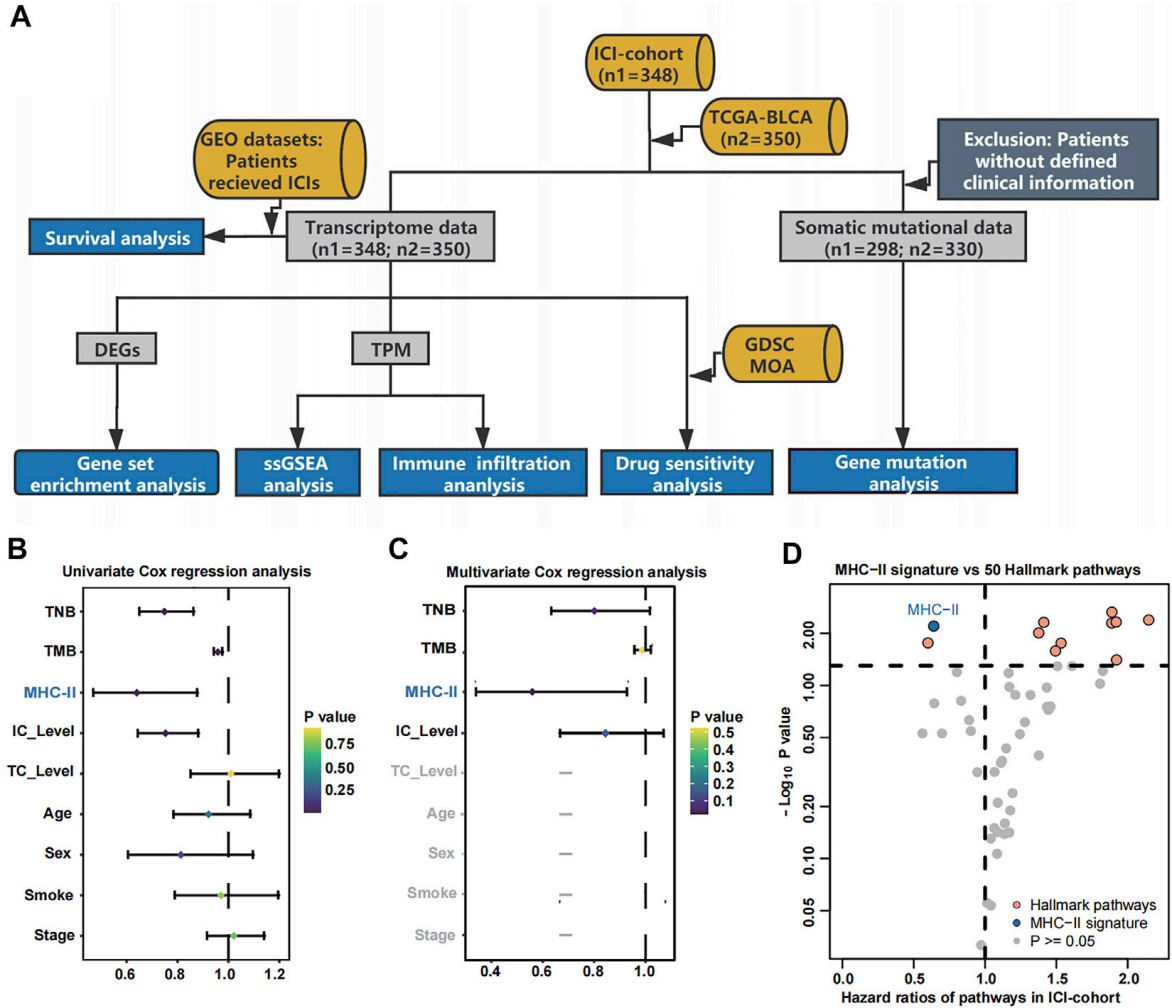
Cox proportional hazards regression analysis results for the ICI-cohort and flowchart. **(A)**. Flowchart. We quantified MHC-II signature by using the ssGSEA method to calculate the MHC-II signature score, then divided the patients into MHC-H and MHC-L groups within the ICI-cohort (*n* = 348) and the TCGA-BLCA cohort (*n* = 350). Based on these categories, patients with different MHC-II signature levels were comprehensively analyzed and classified by their immunome, transcriptome, metabolome, genome and drugome data. We also collected the transcription and survival data of two GEO cohorts treated with immunotherapy to verify the predictive value of the MHC-II signature. MHC-H: MHC-II signature score high; MHC-L: MHC-II signature score low. **(B)** The forest plot displays the results of a univariate analysis. Variables with a Cox P value less than 0.05 are MHC-II, TMB, TNB and IC Level. MHC-II: MHC-II signature score. The signature scores of the gene sets were calculated using the ssGSEA algorithm. **(C)** The forest plot displays the results of a multivariate analysis. Only MHC-II signature was an independent and favorable predictor of bladder cancer patients treated with ICIs (P < 0.05). **(D)** The volcano plot displays the results of a univariate analysis between MHC-II signature score (blue) and 50 hallmark pathways scores (orange and gray). Gray dots indicate pathways with a Cox P value less than 0.05. The hazard ratio [HR] indicates protective (HR < 1) or risk (HR > 1) factors. The horizontal dashed line indicates *p* = 0.05. The gene sets of 50 hallmark pathways were obtained from the MSigDB. Orange dots from left to right indicate the following pathways: spermatogenesis, angiogenesis, coagulation, UV response DN, xenobiotic metabolism, TGFβ signaling, hypoxia, wntβ catenin signaling, reactive oxygen species pathway and p53 pathway.

As shown in the flowchart of Figure 1A, patients with different MHC-II signature levels were comprehensively analyzed based on their immunome, transcriptome, metabolome, genome and drugome data (See in Materials and methods). To access the relationship between MHC-II signature and the prognosis of bladder cancer patients treated with ICIs, univariate Cox regression analysis and multivariate Cox regression analysis were performed. The results showed that only MHC-II signature was an independent and protective factor in the efficacy of ICIs (hazard ratio [HR] = 0.64, 95% confidence interval [CI], 0.34 to 0.93, P < 0.01) (Figures 1B,C). Also, comparing to 50 Hallmark gene pathways signatures (Supplementary Table S2), the MHC-II signature still showed a comparable, favorable predictive value (Figure 1D).

**FIGURE 2:**
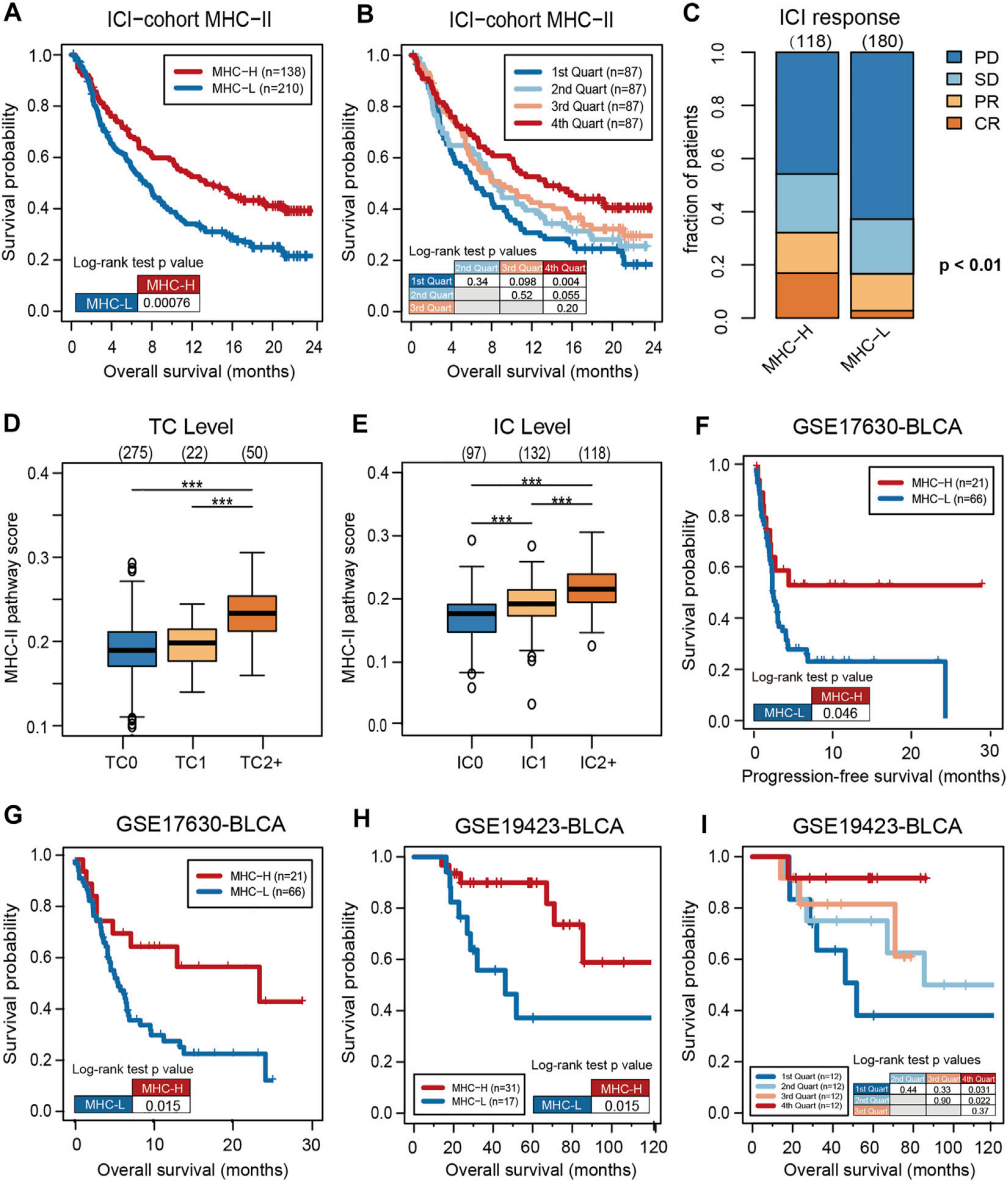
High MHC-II signature predicts the favorable prognosis and immune response of ICIs. **(A)** The KM analysis was performed on MHC-H (*n* = 138) and MHC-L (*n* = 210) groups in the ICI-cohort. The overall survival (OS) of the MHC-H group was significantly longer than that of MHC-L group (hazard ratio [HR] = 0.63, 95% confidence interval [CI]: 0.49–0.82, log rank test P = 7.60e-04). **(B)** MHC-II signature quartiles were also significantly associated with OS. **(C)** MHC-II signature was highly correlated with immune response, particularly with complete response (CR) (two sided Fisher’s exact test, P < 0.01; *n* = 298; The response status of 50 cases was not reported). PD, progressive disease; SD, stable disease; PR, partial response. **(D,E)** MHC-II signature was highly correlated with the expression of PD-L1, including TC levels **(D)** and IC levels **(E)** (all P < 0.001). Tumor tissue samples were scored *via* immunohistochemistry (IHC) for PDL1 expression on tumor cells (TC) and tumor-infiltrating immune cells (IC), respectively. Specimens were scored as IHC TC0, TC1, TC2, or TC3 if <1%, ≥1% but <5%, ≥5% but <50%, or ≥50% of TC were PD-L1 positive, respectively. Specimens were scored as IHC IC0, IC1, IC2, or IC3 if <1%, ≥1% but <5%, ≥5% but <10%, or ≥10% of IC were PD-L1 positive, respectively. Tumor-infiltrating immune cells included macrophages, dendritic cells and lymphocytes. *P < 0.05, **P < 0.01, ***P < 0.001, and ****P < 0.0001, ns, not significant. **(F)** Validation of the GSE17630−BLCA cohort (*n* = 87) treated with ICIs showed that the PFS of the MHC-H group was significantly longer than that of the MHC-L group (HR = 0.50, 95% CI: 0.28–0.89, log rank test P = 0.046). **(G)**. Validation of the GSE17630−BLCA cohort (*n* = 87) treated with ICIs showed that the OS of the MHC-H group was significantly longer than that of the MHC-L group (HR = 0.43, 95% CI: 0.24–0.76, log rank test P = 0.015). **(H)** Validation of the GSE19423-BLCA cohort (*n* = 48) treated with ICIs showed that the OS of the MHC-H group was significantly longer than that of the MHC-L group (HR = 0.32, 95% CI: 0.11–0.94, log rank test P = 0.015). **(I)** Validation of the GSE19423-BLCA cohort treated with ICIs showed that MHC-II signature quartiles were also significantly correlated with OS.

To verify the predictive value of the MHC-II signature, KM analysis was performed on MHC-H and MHC-L groups in the ICI-cohort. Consistent with the results of the multivariate analysis, the overall survival (OS) of the MHC-H group was significantly longer than that of the MHC-L group (HR = 0.63, 95% CI: 0.49–0.82, log rank test P = 7.60e-04) (Figure 2A). Further, MHC-II signature quartiles were significantly related to OS (Figure 2B). MHC-II signature was also highly correlated with response, particularly with complete response, and with PD-L1 expression on tumor cells (TC level) and immune cells (IC level) (Figures 2C–E).

Further validation in the GEO bladder cancer cohort treated with anti-PD-1 inhibitors showed that the PFS and OS of the MHC-H group was significantly longer than that of the MHC-L group (PFS, HR = 0.50, 95% CI: 0.28–0.89, log rank test P = 0.046; OS, HR = 0.43, 95% CI: 0.24–0.76, log rank test P = 0.015) (Figures 2F,G). Meanwhile, bladder cancer patients from the GEO cohort treated with BCG immunotherapy also presented that MHC-H signature is significantly associated with OS (MHC-H, HR = 0.32, 95% CI: 0.11–0.94, log rank test P = 0.015) (Figures 2H,I).

### MHC-II Signature as a Predictive Biomarker is Superior to Tumor Mutation Burden and inferior to Tumor Neoantigen Burden

**FIGURE 3:**
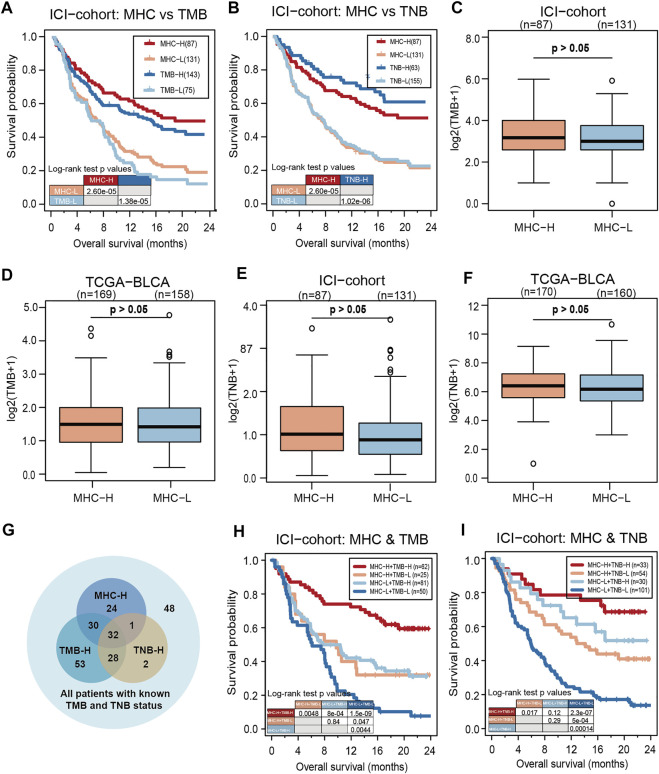
MHC-II signature is superior to TMB and slightly inferior to TNB as predictive biomarkers. **(A)** KM analysis showed that MHC-II signature was superior to TMB in the ICI-cohort (MHC-II signature: HR = 0.47, 95% CI: 0.34–0.65, P = 2.60e-05; TMB: HR = 0.49, 95% CI: 0.34–0.70, P = 1.38e-05). **(B)** KM analysis showed that MHC-II signature was slightly inferior to TNB in the ICI-cohort (MHC-II signature: HR = 0.47, 95% CI: 0.34–0.65, P = 2.60e-05; TNB: HR = 0.35, 95% CI: 0.25–0.49, P = 1.02e-06). **(C,D)** Boxplots show that there was no significant relationship between MHC-II signature and TMB in either the ICI-cohort **(C)** or the TCGA-BLCA cohort **(D)** (Wilcoxon Test, all P > 0.05). E-F. Boxplots showed that there was no significant relationship between MHC-II signature and TNB in either the ICI-cohort **(E)** or the TCGA-BLCA cohort **(F)** (wilcoxon test, all P > 0.05). **(G)** Venn diagram showed the distribution in the MHC-H and MHC-L groups of patients with known TMB and TNB status in the ICI-cohort (*n* = 218). **(H)** KM curves show the comparison of MHC-H + TMB-H, MHC-H + TMB-L, MHC-L + TMB-H and MHC-L + TMB-L in ICI-cohort (*n* = 218). **(I)** KM curves show the comparison of MHC-H + TNB-H, MHC-H + TNB-L, MHC-L + TNB-H and MHC-L + TNB-L in the ICI-cohort (*n* = 218).

To evaluate the predictive performance of MHC-II signature on ICIs, we compared MHC-II signature with reliable predictive markers of bladder cancer such as TMB and TNB. The result showed that MHC-II signature was superior to TMB in its prediction capabilities (MHC-II signature: HR = 0.47, 95% CI: 0.34–0.65, P = 2.60e-05; TMB: HR = 0.49, 95% CI: 0.34–0.70, P = 1.38e-05) (Figure 3A), but inferior to TNB (MHC-II signature: HR = 0.47, 95% CI: 0.34–0.65, P = 2.60e-05; TNB: HR = 0.35, 95% CI: 0.25–0.49, P = 1.02e-06) (Figure 3B). Based on the fact that enhanced tumor immunogenicity predicts improved response to ICIs, we next compared the TMB and TNB levels between MHC-H and MHC-L tumors. Ultimately, in both the ICI-cohort and the TCGA-BLCA cohort, there was no significant relationship between MHC-II signature and TMB or TNB (Figures 3C–F). This indicates that other mechanisms may exist that correlate with MHC-II signature increasing tumor immunogenicity in MHC-H tumors.

Clinically, despite the limited predictive abilities of TMB and TNB in evaluating the effectiveness of ICIs, there are still quite a few patients with TMB-L or TNB-L who remain unsure as to whether ICIs will be an effective course of treatment (Researchers Strive to Refine, 2021). Therefore, we included patients with TMB and TNB information in the ICI-cohort for subsequent analysis (*n* = 218) (Figure 3G). Among TMB-L and TNB-L patients, the MHC-H group could still clearly identify patients with longer OS compared with the MHC-L group. Furthermore, either TMB or TNB combined with MHC-II signature could predict better prognosis of ICIs than TMB or TNB alone (Figures 3H,I). All the above results suggest that MHC-II signature is comparable and compatible with TMB or TNB as a predictive biomarker.

### MHC-II Signature Shows Correlation With Immune Cell Infiltration and Anti-Tumor Immunity.

**FIGURE 4:**
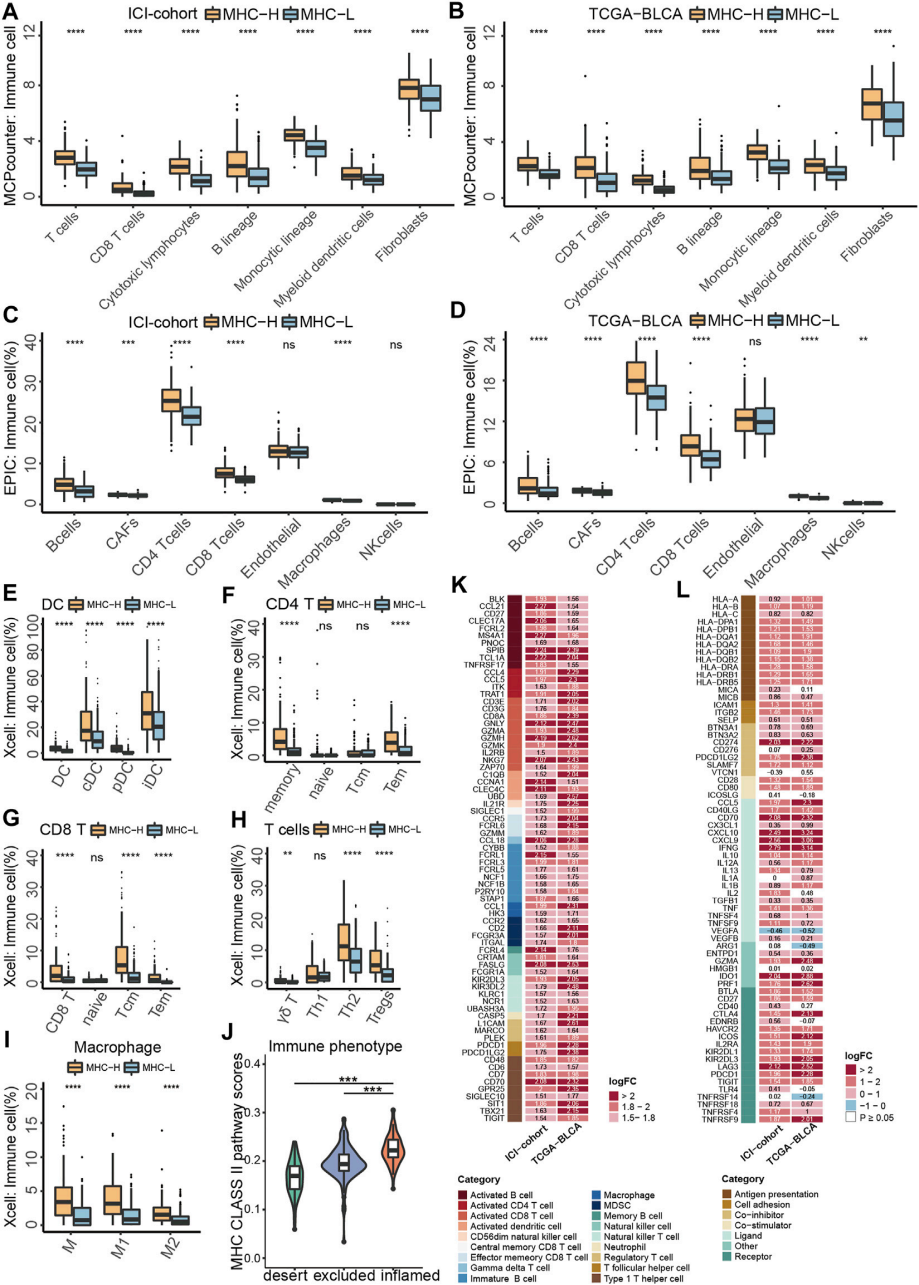
MHC-II signature shows correlation with immune cell infiltration and anti-tumor immunity. **(A,B)** MCP-counter analyses quantifying immune cells and stromal cells in MHC-H and MHC-L groups in ICI-cohort **(A)** and TCGA-BLCA cohort **(B) (C,D)** EPIC analyses quantifying the infiltration ratio of immune cells of the MHC-H and MHC-L groups in the ICI-cohort **(C)** and TCGA-BLCA cohort **(D) (E–I)** xCell analyses estimate the abundance scores of 64 kinds of immune cells in the MHC-H and MHC-L groups in the ICI-cohort. Dendritic cells **(E)** include conventional dendritic cells (cDCs), plasmacytoid dendritic cells (pDCs) and immature dendritic cells (iDCs); CD4+T cells **(F)** include CD4+memory T cells, CD4+naive T cells, CD4^+^ central memory T cells and CD4^+^ effector memory T cells; CD8+T cells **(G)** include CD8^+^ naive T cells, CD8^+^ T central memory T cells and CD8^+^ T effector memory T cells; other types of T cells **(H)** include T cell gamma delta cells (Tgd), T helper 2 cells (Th2) and regulatory cells (Tregs). Macrophages **(I)** include M1 macrophages and M2 macrophages. **(J)** Violin plot showed the distribution of tumor immunophenotype in the MHC-H and MHC-L groups of the ICI-cohort. **(K)** Heatmap showing the significant difference in the average expression of immune cell-related genes (logFC ≥ 1.5 and P < 0.01) between the MHC-H and MHC-L groups of the ICI-cohort and TCGA-BLCA cohort. Genes which correspond to the same cell type are indicated by the same color. From left to right are the name of the gene, the cell type corresponding to the gene, and the direction of change in gene expression. Red in the right rectangle indicates up-regulation, and blue indicates down-regulation. The logFC value was marked in the right rectangle. **(L)** Heatmap showing the significant difference in the average expression of immune-related genes between the MHC-H and MHC-L groups in the ICI-cohort and the TCGA-BLCA cohort. Genes of the same category were indicated by the same color. From left to right are the name of the gene, gene function, and the direction of change in gene expression. Red in the right rectangle indicates up-regulation, blue indicates down-regulation, and white indicates that the result was not significant (P > 0.05). The logFC value was marked in the right rectangle. **(A−J)** *P < 0.05, **P < 0.01, ***P < 0.001, and ****P < 0.0001, ns, not significant.

To identify tumor microenvironment (TME) characteristics of patients with MHC-H, we integrated a series of algorithms to compare immune cell infiltration between the MHC-H and MHC-L groups. Considering that MCP-counter (Becht et al., 2016) is a reliable algorithm for comparisons between samples, we first used the MCP-counter algorithm to compare immune cells infiltration between two groups in the ICI-cohort. The results showed that immune cells were highly infiltrated in MHC-H tumors (all P < 1e-04, Figure 4A). These results were verified in the TCGA-BLCA cohort (all P < 1e-04, Figure 4B). In addition, the EPIC algorithm (Racle and Gfeller, 2020) was performed to ensure the stability of the results. The results consistently showed that B cells, activated fibroblasts (CAFs), CD4^+^ T cells, CD8^+^ T cells, and macrophages were all highly infiltrated in MHC-H tumors (all P < 0.001, Figure 4C). These results were also validated in the TCGA-BLCA cohort (all P < 1e-04, Figure 4D). Next, we used xCell (Aran et al., 2017) to further compare the specific subtypes of immune cells with infiltration differences between the two groups. Considering that MHC-II molecules are mainly expressed by professional antigen presenting cells (pAPCs) such as dendritic cells (DCs) and mainly present exogenous peptide antigens to CD4^+^ T cells (Wang, 2001), thus, we focused on DCs and CD4^+^ T cells. Results of the xCell analyses supported that DCs, CD4^+^ T cells, CD8^+^ T cells, helper T cells, and macrophages (especially M1 macrophages) were highly infiltrated in MHC-H tumors (all *p* < 0.01, Figures 4E–I). Notably, there is more increase in Th2 and Treg cells than for Th1 cells, which may be attributed to the complexity of immune infiltration, which allows for immune cells to be either suppressed or activated in response to antigens (Figure 4H). Among the three immunological phenotypes of solid tumors (immune inflamed, immune excluded, and immune desert), previous evidence has shown that inflamed tumors are the most responsive to ICIs (Hegde et al., 2016). Consistent with this, the MHC-II signature score was lowest in immune desert tumors and highest in immune inflamed tumors (Figure 4J).

Besides, MHC-H tumors were significantly associated with upregulated expression of genes related to activated dendritic cells (CCNA1 and CLEC4C), activated CD4^+^ T cells (CCL4 and CCL5), and activated CD8^+^ T cells (GNLY, GZMH and NKG7) in both the ICI-cohort and the TCGA-BLCA cohort (logFC > 1, all P < 0.01, Figure 4K). Additionally, the analysis of immune-related genes showed that almost all genes related to antigen presentation and immune activation were enriched in the MHC-H group of both the ICI-cohort and the TCGA-BLCA cohort, especially those related to cross talk between immune cells (IFNG, CXCL9 and CXCL10) as well as ICIs therapy (LAG3, IDO1, CD70 and CD274) (log FC > 2, P < 0.05, Figure 4L).

Taken together, the results above indicate that tumors with MHC-H have an immune activation microenvironment characterized by increased infiltration of activated dendritic cells, CD4^+^ T cells, and CD8^+^ T cells. This kind of microenvironment enhances anti-tumor immunity and may contribute to MHC-II signature predictive value for the degree of sensitivity to ICI therapy.

### Transcriptome Traits Related to MHC-II Signature

**FIGURE 5:**
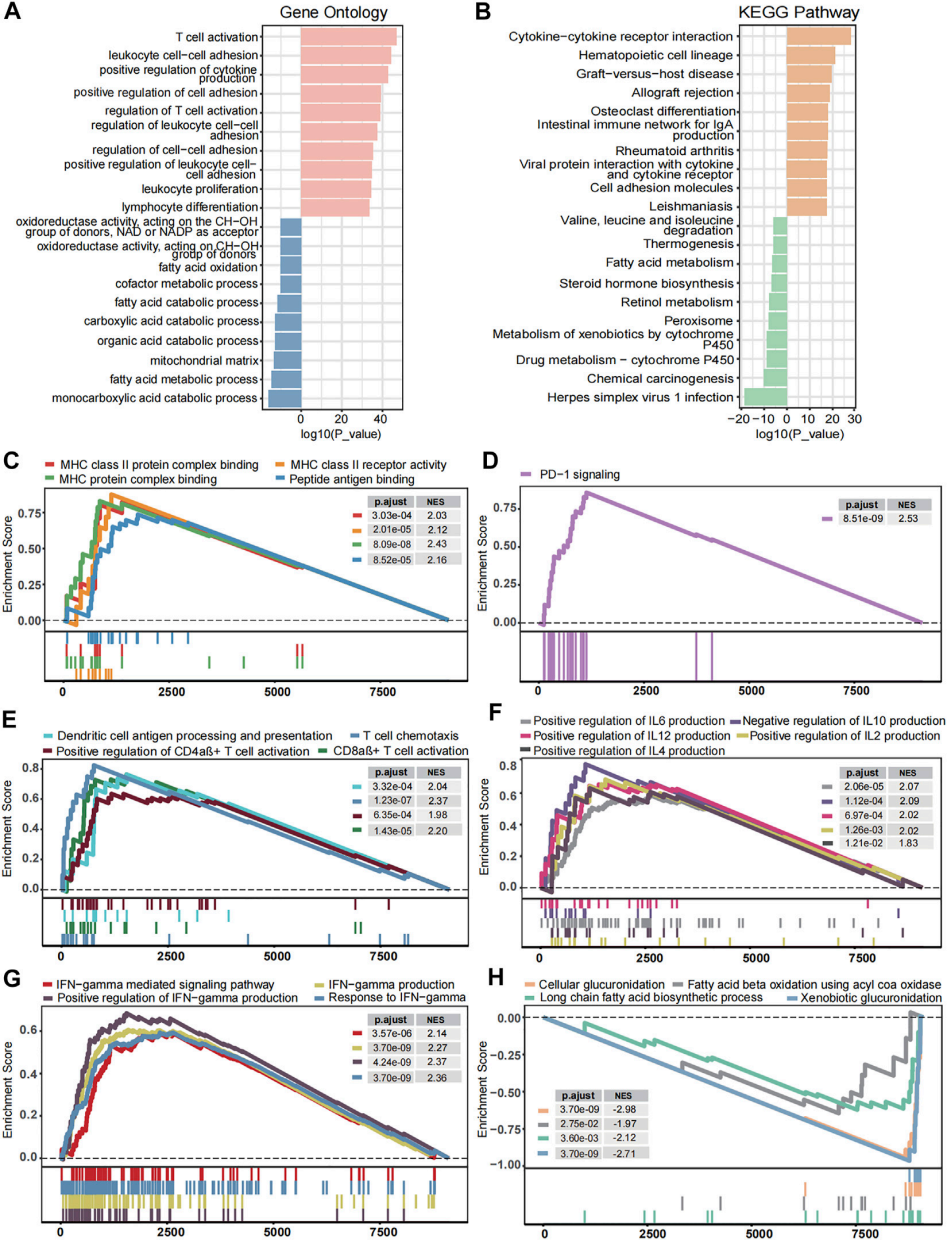
Transcriptome traits related to MHC-II signature in the ICI-cohort. **(A,B)**. Gene Ontology enrichment **(A)** and KEGG enrichment analyses **(B)** show that gene sets up-regulated in MHC-H tumors were enriched in the immune activation process, while those overexpressed in MHC-L tumors were enriched in fatty acid metabolism and steroid hormone biosynthesis. The top ten genes per set are shown (ranked by single-gene *p* value, GO: red: high, blue: low; KEGG: orange: high, green: low). **(C–H)** The GSEA analysis shows the key pathways of enrichment in the MHC-H (up) and MHC-L (down) groups. Antigen processing and presentation **(C)**, PD−1 signaling **(D)**, immune cell activation **(E)**, cytokine production **(F)**, and IFN−gamma **(G)** related pathways were significantly up-regulated in the MHC-H group, while fatty acid metabolism and glucuronidation **(H)** were significantly up-regulated in the MHC-L group. The *x*-axis represents the ranking of genes in the rank lists.

To discover the underlying mechanism that contributes to MHC-II signature’s valuable predictive power for BC, we comprehensively explored transcriptomic traits related to MHC-II signature. Gene Ontology enrichment and KEGG enrichment analyses showed that gene sets upregulated in MHC-H tumors were consistently enriched in the immune activation process, while those overexpressed in MHC-L tumors were enriched in fatty acid metabolism and steroid hormone biosynthesis (Figures 5A,B). Further GSEA analysis showed similar results. Immune response-related pathways were significantly upregulated in the MHC-H group (adjust P < 0.05, Figures 5C–G). These pathways include antigen processing and presentation (especially *via* MHC-II), PD−1 signaling, immune cell (DCs, CD4αβ+ T cells, CD8αβ+ T cells) activation, cytokine production, and IFN−gamma related pathways. In contrast, the functional pathways related to fatty acid metabolism and glucuronidation were significantly down-regulated in the MHC-H group (adjust P < 0.05, Figure 5H). All the results above could be clearly verified in the TCGA-BLCA cohort (adjust P < 0.05, Supplementary Figures S1A–F). Thus, we reasonably infer that higher antigen presentation and immune activation as well as lower fatty acid metabolism and glucuronidation in MHC-H tumors may help explain the predictive value for ICIs treatment sensitivity.

### Genomic Landscape Related to MHC-II Signature

**FIGURE 6:**
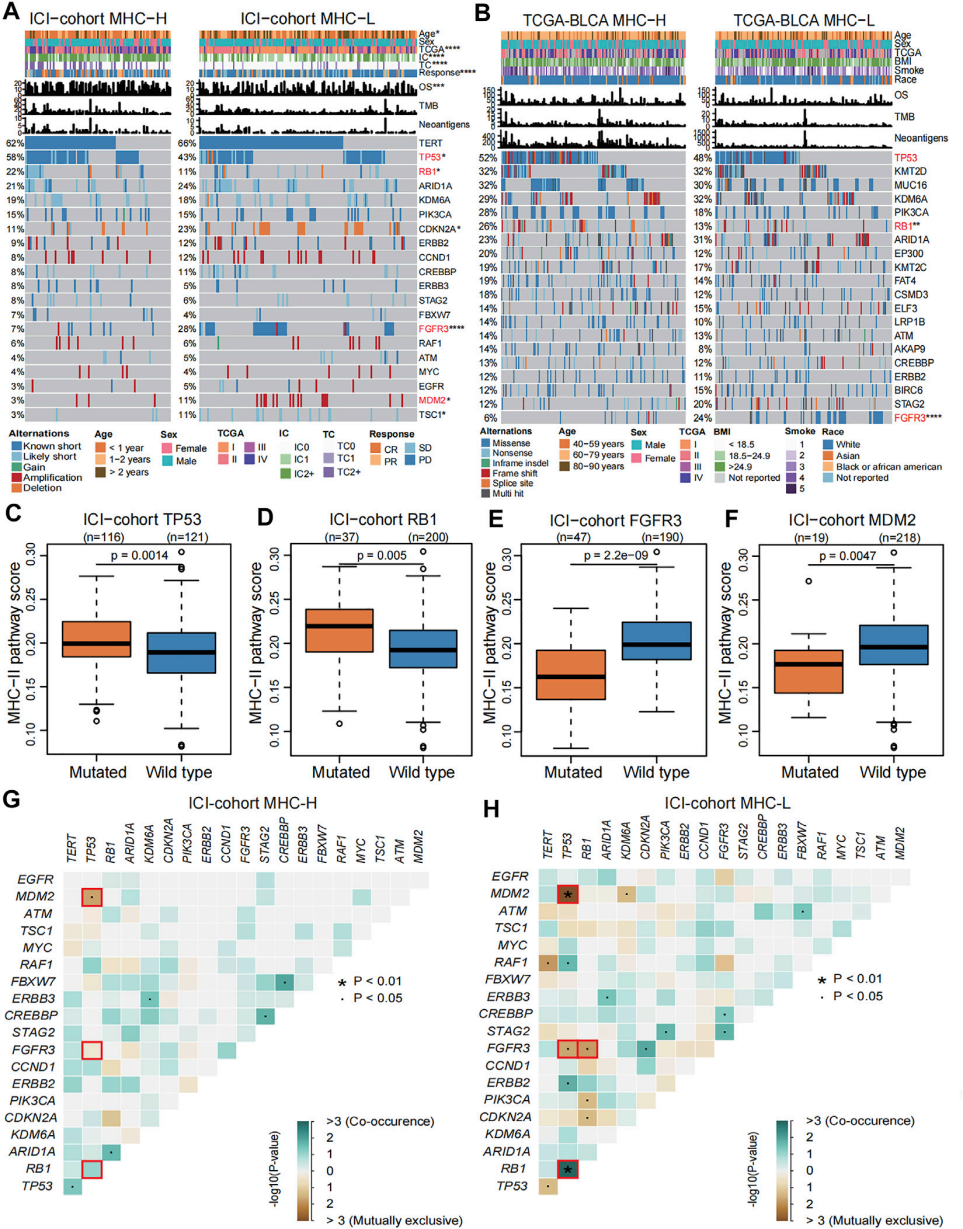
Genomic landscape related to MHC-II signature. **(A)** Comparison of the differences in the mutation status in the top 20 genes with mutations, diagnosis years (age), sex, TCGA stage, PD-L1 expression (IC level and TC level), immune response, OS, TMB and TNB between MHC-H (left) and MHC-L groups (right) in the ICI-cohort. Genes were ranked by mutation frequency (left panel). The mutation frequencies of TP53, RB1, FGFR3, and MDM2 genes were significantly different between two groups, which are marked with red font. *P < 0.05, **P < 0.01, ***P < 0.001, and ****P < 0.0001. **(B)** Comparison of the differences in the mutation status in the top 20 genes with mutations, age, sex, TCGA stage, BMI, smoking status, race, OS, TMB and TNB between the MHC-H (left) and MHC-L groups (right) in TCGA-BLCA cohort. Genes were ranked by mutation frequency (left panel). The mutation frequencies of TP53, RB1, and FGFR3 genes were significantly different between two groups, which are marked with red font. *P < 0.05, **P < 0.01, ***P < 0.001, and ****P < 0.0001. C-F. Boxplots show that TP53 **(C)** and RB1 **(D)** gene mutations were significantly correlated with high MHC-II signature in the ICI-cohort (Mann Whitney U test, P = 0.0014, P = 0.005, respectively), while FGFR3 **(E)** and MDM2 **(F)** gene mutations are significantly correlated with low MHC-II signature in the ICI-cohort (Mann Whitney U test, P = 2.2e-09, P = 0.0047, respectively). G-H. Concurrence (blue) and mutual exclusion (brown) between high frequency mutation genes (the top 20 genes with mutations) in MHC-H **(G)** and MHC-L **(H)** groups in the ICI-cohort. ∙P < 0.05, *P < 0.01.

To describe the genomic landscape related to the MHC-II signature, we compared the differences in tumor-intrinsic genomic alterations and clinical phenotypes between the MHC-H and MHC-L groups in the ICI-cohort and the TCGA-BLCA cohort. Our results show that, in comparison with the MHC-L group, the MHC-H group had higher PD-L1 expression, better immune response and prognosis, and higher mutation frequencies of both TP53 (ICI-cohort: 58 vs. 43%, P = 0.0014; TCGA-BLCA: 52 vs. 48%, P = 0.099) and RB1 (ICI-cohort: 22 vs. 11%, P = 0.005; TCGA-BLCA: 26 vs. 13%, P = 0.0012). Despite this, there was no significant difference in TP53 mutation frequency between the two groups in the TCGA-BLCA cohort (P = 0.099). In the MHC-L group, the mutation frequencies of FGFR3 (ICI-cohort: 28 vs. 7%, P = 2.2e-09; TCGA-BLCA: 24 vs. 8%, P = 2.7e-06) and MDM2 (ICI-cohort: 11 vs. 3%, P = 0.0047) were significantly higher than those of the MHC-H group (Figures 6A,B). Consistent with this, in the ICI-cohort, the MHC-II signature score was significantly higher in the TP53 or RB1 mutated groups compared to the wild type groups, and the MHC-II signature score was lower in FGFR3 or MDM2 mutated groups than the wild type groups (all P < 0.01, Figures 6C–F). The results above were strongly validated by the TCGA-BLCA cohort (Figure 6B, Supplementary Figures S2A–C). Additionally, among the clinical characteristics of the ICI-cohort, the tumor TCGA stage was significantly correlated with the MHC-II signature score (Supplementary Figure S2D).

We further explored the co-occurrence and mutual exclusion between high-frequency mutant genes and found that TP53 gene mutations tended to be concurrent with RB1 gene mutations. At the same time, the TP53 mutations were usually mutually exclusive with FGFR3 gene mutations in both MHC-H and MHC-L tumors (Figures 6F,G, Supplementary Figures S2E, F). For the MHC-L tumors, RB1 gene mutations also tended to be mutually exclusive with FGFR3 gene mutations (Figure 6H, Supplementary Figure S2F). TP53 gene mutations and MDM2 gene mutations tended to be mutually exclusive in the ICI-cohort, which is consistent with previous research (Soussi and Kroemer, 2018) (Figures 6G,H). In the MHC-L group of the TCGA-BLCA cohort, FGFR3 gene mutations were also highly concurrent with PIK3CA gene mutations (Supplementary Figure S2F).

To further explore the relationship between MHC-II signature and the FGFR3, TP53 and RB1 gene mutations, we compared the difference in mutation sites of three genes from the MHC-H and MHC-L groups in the TCGA-BLCA cohort. We found that there was a frameshift deletion (p.H791Tfs*29) in the catalytic domain of the protein Tyrosine Kinase in the FGFR3 gene of MHC-L tumors (Supplementary Figure S3A). Although most TP53 mutations were located in the DNA binding domain, we found an in-frame deletion (p. e336_R337de) in the P53 tetramerization motif domain in MHC-H tumors (Supplementary Figure S3B). In the RB1 gene, somatic mutations were evenly distributed without any annotated functional hotspot mutations from 3D hotspots (Gao et al., 2017) (Supplementary Figure S3C).

### Role of MHC-II Signature in Drug Sensitivity Prediction

**FIGURE 7:**
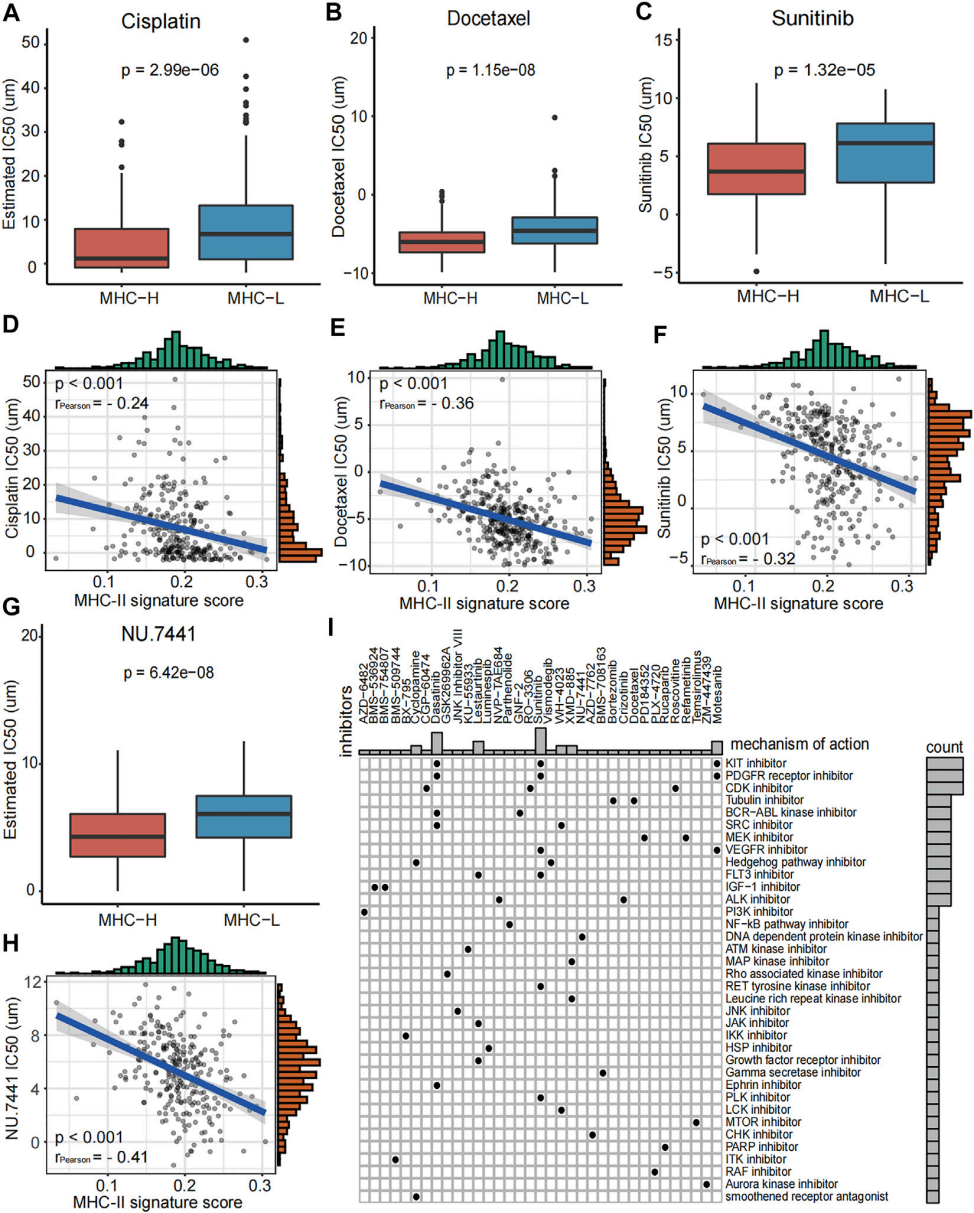
Role of MHC-II signature in drug sensitivity prediction in the ICI-cohort. **(A–H)** Boxplots show that the IC50 values of Cisplatin **(A)**, Docetaxel **(B)**, Sunitinib **(C)** and NU.7441 **(G)** were significantly lower in the MHC-H group compared to the MHC-L group (Wilcoxon.test, P = 2.99e−06, P = 1.15e−08, P = 1.32e−5, P = 6.42e−08, respectively). And IC50 values of Cisplatin **(D)**, Docetaxel **(E)**, Sunitinib **(F)** and NU.7441 **(H)** were negatively correlated with MHC-II signature score (Pearson test, r_Pearson_ = −0.24, r_Pearson_ = −0.36, r_Pearson_ = −0.32, r_Pearson_ = −0.41, respectively. All P < 0.001. **(I)** Heatmap shows the MoA (row) shared by each compound (column, *n* = 35) in the ICI-cohort. The MoA is sorted according to the number of compounds sharing the MoA, displayed in the heatmap.

In order to better guide the clinical treatment of BC patients, we performed a series of analysis as described in methods. The IC50 values of Cisplatin, Docetaxel, Sunitinib, and NU.7441 in the MHC-H group were significantly lower than those of the MHC-L group, and all of these values were significantly correlated with the MHC-II signature score (all P < 0.001, Figures 7A–H). The results indicate that patients with MHC-H will have increased sensitivity when treating bladder cancer with Cisplatin, Docetaxel, Sunitinib, and NU.7441. Furthermore, to explore the potential mechanism of the increased drug sensitivity with MHC-H tumors, the MoA analysis was performed. We found that combining immunotherapy with KIT inhibitors (such as Dasatinib and Sunitinib), PDGFR receptor inhibitor, CDK inhibitor or Tubulin inhibitor may benefit BC patients with high MHC-II signature (Figure 7I). All the results above were definitively verified by the TCGA-BLCA cohort (Supplementary Figure S4).

## Discussion

Despite increasing evidence supports that CD4^+^ T cells involved in MHC-II-mediated tumor killing potentially play a key role in anti-tumor immunity (Nagasaki et al., 2020; Oh et al., 2020; Sacher et al., 2020), studies focusing on the association between MHC-II signature and immune response or the prognosis of malignant tumors has not been thoroughly researched and reported. To explore the relationship between MHC-II signature and ICIs therapy, we collected an ICI-cohort, a TCGA-BLCA cohort and two GEO cohorts treated with ICIs. Additionally, comprehensive analyses of immunome, transcriptome, metabolome, genome, and drugome were performed to characterize and dissect the association of MHC-II signature and tumor immunotherapy.

Firstly, we identified that MHC-II signature is a favorable and independent predictor of bladder cancer prognosis after immunotherapy, and its predictive value statistically be superior to TMB. Notably, in both the ICI-cohort and the TCGA-BLCA cohort, there was no significant relationship between MHC-II signature and TMB or TNB. This indicates that there may exist other mechanisms correlated with MHC-II signature’s ability to increase tumor immunogenicity in MHC-H tumors. In clinical instances, a number of patients with TMB-H or TNB-H had poor prognoses after immunotherapy that brought challenges to making therapeutic choices (Researchers Strive to Refine, 2021). Fortunately, for patients with TMB-H or TNB-H, we identified that the prognosis of the MHC-H group was significantly better than that of the MHC-L group. We also found that the combination of TMB or TNB with MHC-II signature improved the predictive value of prognosis for ICIs therapy.

Secondary, we determined that the tumors with high MHC-II signature were characterized by increased immune cells infiltration and enhanced anti-tumor immunity. The majority of immune cells had significantly higher infiltration in the MHC-H group than in the MHC-L group. A previous study showed that DCs could present tumor antigens to T cells within the tumor itself; however, this intratumoral performance depends on DC-T cell crosstalk: the T cell reaction depends on IL-12 production, while DC reacts to IFN-γ produced by T cells (Garris et al., 2018). In addition to the reaction of DCs to T cells in primary tumors, there is mounting evidence that CD4^+^ T cells play a major role in immune response (Nagasaki et al., 2020). A recent study has found that CD4 T cells representing an alternative/synergistic population to classical CD8 T cells and the cytolytic activity of CD4 T cells against tumors requires MHC-II expression in cancer cells (Cachot et al., 2021). Previous study also found that MHC class II on B cells contributes to the development, differentiation, and effector functions of CD4^+^ T cells in response to T cell-dependent antigen (Shimoda et al., 19502006). Collectively, these studies are consistent with our finding of increased immune cell infiltration in the MHC-H group and explain the better prognosis of patients with high MHC-II signature after immunotherapy. We also found that, compared with the MHC-L group, the heterogeneous states of DCs expressing CCNA1 and CLEC4C, CD4^+^ T cells expressing CCL4 and CCL5, CD8^+^ T cells expressing cytolytic effector molecules (GZMH and GZMK), and other granule-associated proteins (GNLY) might play a role in killing target cells in the MHC-H group. These all indicate that the level of MHC-II signature is connected to these effector cells, thus providing a direction for future work in identifying BC patients who are more responsive to immunotherapy. Consistent with this, pathway analysis showed that pathways related to the immune activation process were significantly enriched in the MHC-H group. On the contrary, the functional pathways related to fatty acid metabolism and glucuronidation were significantly enriched in the MHC-L group. Since activated T cells require increased glucose and fatty acids to proliferate and mediate effector functions (Byersdorfer et al., 2013), we hypothesized that enriched fatty acid metabolism in MHC-L tumors may lead to the depletion of T cells in the TME, thus reducing the sensitivity to immunotherapy. A recent study reported that glucuronidation might be linked to tumor recurrence and metastasis (Wang et al., 2019a). This may partly explain the poor prognosis of patients with MHC-L tumors after immunotherapy.

Third, we comprehensively described the mutation landscape of tumors with different MHC-II signature levels within the genome. Cancer is driven by somatic mutations that result in the generation of neoantigens. The presentation of these novel peptides depend on patients’ MHC genotype, thus this selective advantage of somatic mutations promises to be counterbalanced by the immune system (Vogelstein et al., 2013). We can thus reasonably infer that a distinct MHC-II signature is characterized by a distinct mutation landscape. FGFR3 gene has always been related to the occurrence of BC, with Food and Drug Administration (FDA) approved FGFR targeting agents used as a treatment for BC (Nadal and Bellmunt, 2019). Prior studies have demonstrated that FGFR3-mutant urothelial cancers were associated with a decrease in T cell infiltration and poor response to ICI treatments (Wang et al., 2019b), which is consistent with our results. Considering its inverse relationship with anti-tumor immune response and the relationship to the phenotype of lymphocyte exclusion (Sweis et al., 2016), it is a reasonable to expect MHC-L tumors with low immune infiltration to be enriched by FGFR3 gene mutations. Importantly, these results further explain why patients with FGFR3 wild type gene were more suitable for immunotherapy than patients with FGFR3 mutant gene in clinical trials (Kacew and Sweis, 2020). MDM2, a negative regulator of the tumor suppressor protein p53, may promote an anti-tumor immune microenvironment *via* p53 and immune cell activation (Momand et al., 1998). It has been reported that an MDM2 antagonist, APG-115, was found to decrease the immunosuppressive M2 macrophage population and increase the proinflammatory M1 macrophage polarization in the spleen of mice treated with APG-115 (Fang et al., 2019), which is consistent with our results. Moreover, we found that the MHC-H group with better prognosis had higher mutation frequencies of TP53 and RB1 genes, which is consistent with the conclusion of one of our previous studies that TP53 mutation may predict the favorable prognosis of BC patients treated with ICIs (Lyu et al., 2020). By analyzing the association between high-frequency mutation genes, we found that the FGFR3 gene mutation tended to be mutually exclusive with TP53 and RB1 gene mutations. This further confirms the distinct mutation landscapes of tumors with different MHC-II signature levels.

Finally, we identified a significant increase in drug sensitivity to cisplatin, docetaxel, sunitinib and NU.7441 in the MHC-H group. Cisplatin and docetaxel are commonly used as first-line treatment drugs for BC. Sunitinib is a multi-target receptor tyrosine kinase inhibitor that can inhibit VEGFR2 and PDGFR. Studies have reported that cisplatin and sunitinib could benefit NSCLC when combined with immunotherapy, by increasing the expression of PD-L1 in tumors (Fournel et al., 2019; Li et al., 2021). NU.7441 has been reported to have an impact on the immunobiology of tumor cells and T cells in melanoma. It has been shown to enhance the efficacy of various immunotherapies, such as anti-PD-L1 (Tsai et al., 2017). Combined with our drug sensitivity analysis results, we could reasonably infer that BC patients with MHC-H may benefit from immunotherapy combined with the drugs mentioned above. We also used an MoA analysis to identify other candidate compounds that may be effective to combine with immunotherapy for BC patients with MHC-H. These include KIT inhibitors (dasatinib and sunitinib), PDGFR receptor inhibitors (dasatinib and sunitinib), CDK inhibitor (CGP−60474), and tubulin inhibitor.

**FIGURE 8:**
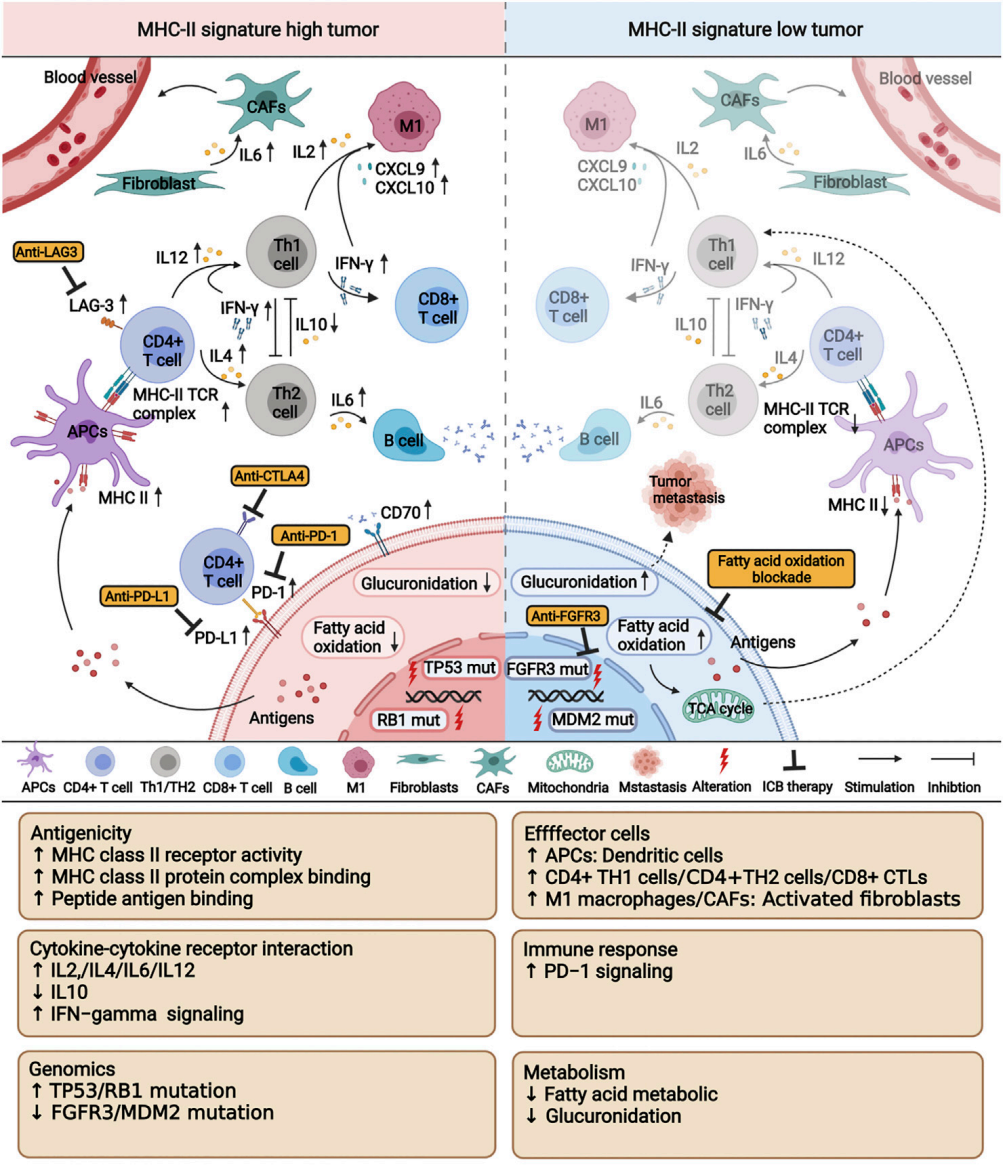
A proposed mechanism underlying the improved efficacy and prognosis in bladder cancer patients with high MHC-II signature after immunotherapy. This picture was created with BioRender.com (https://app.biorender.com/biorender-templates).

Although the comprehensive evaluation of multi-omics data on BC patients has drawn some important conclusions, there are several limitations in this study. First, our results should be further verified in a larger cohort to more precisely define the cutoff values. Second, due to the lack of a BC cohort with expression data after ICIs therapy, we could only verify the relationship between MHC-II signature and the immune microenvironment in the TCGA-BLCA cohort. Third, our analysis only discusses the characteristics of BC patients with high MHC-II signature after ICIs therapy from the perspective of multi-omics. This analysis lacks a comprehensive investigation of the association between MHC-II signature and neoantigens. To tackle this challenge, we have set up a research group working to identify neoantigens related to MHC-II signature. Our research defined the MHC-II signature and introduced the novel finding that MHC-II signature could predict favorable immune response and prognosis of BC treated with ICIs therapy, potentially surpassing the predicative ability of TMB. We also characterized the associations of MHC-II signature and anti-tumor immunity, verifying its predictive value and identifying potential therapeutic targets (Figure 8).

## Data Availability

The datasets presented in this study can be found in online repositories. The names of the repository/repositories and accession number(s) can be found in the article/Supplementary Material.

## Abbreviations

ICI, immune checkpoint inhibitor; BC, bladder cancer; TMB, tumor mutation burden; FGFR3, fibroblast growth factor receptor 3; TGFβ, transforming growth factor β; CTL, cytotoxic T cells; MHC-II, MHC class II; GEO, gene expression omnibus; TCGA-BLCA, the cancer genome atlas bladder cancer; TNB, tumor neoantigen burden; MHC-H, high MHC-II signature score; MHC-L, low MHC-II signature score; NSCLC, non-small cell lung cancer; IC, immune cells; TC, tumor cells; OS, overall survival; SKCM, melanoma; PFS, progression free survival; TME, regulatory cells; pAPCs, professional antigen presenting cells; DCs, dendritic cells; cDCs, conventional dendritic cells; pDCs, plasmacytoid dendritic cells; iDCs, immature dendritic cells; TH2, T helper 2 cells; Tregs, regulatory cells; MOA, mechanism of action; TPM, Transcripts Per Million; ESs, enrichment scores; DGE, differential gene expression; GO, gene ontology; KEGG, kyoto encyclopedia of genes and genomes; FDR, false discovery rate; GSEA, gene set enrichment analysis; MSigDB, the molecular signatures database; GDSC, the genomics of drug sensitivity in cancer.
