# Supplementary material for: MHC-II Signature Correlates With Anti-Tumor Immunity and Predicts anti-PD-L1 Response of Bladder Cancer
Source: Front Cell Dev Biol. 2022 Feb 11;10:757137. doi: 10.3389/fcell.2022.757137 (PMC8873787; doi:10.3389/fcell.2022.757137)
Supplement: Supplementary file 2 [file Presentation1.PDF]

Supplementary figures

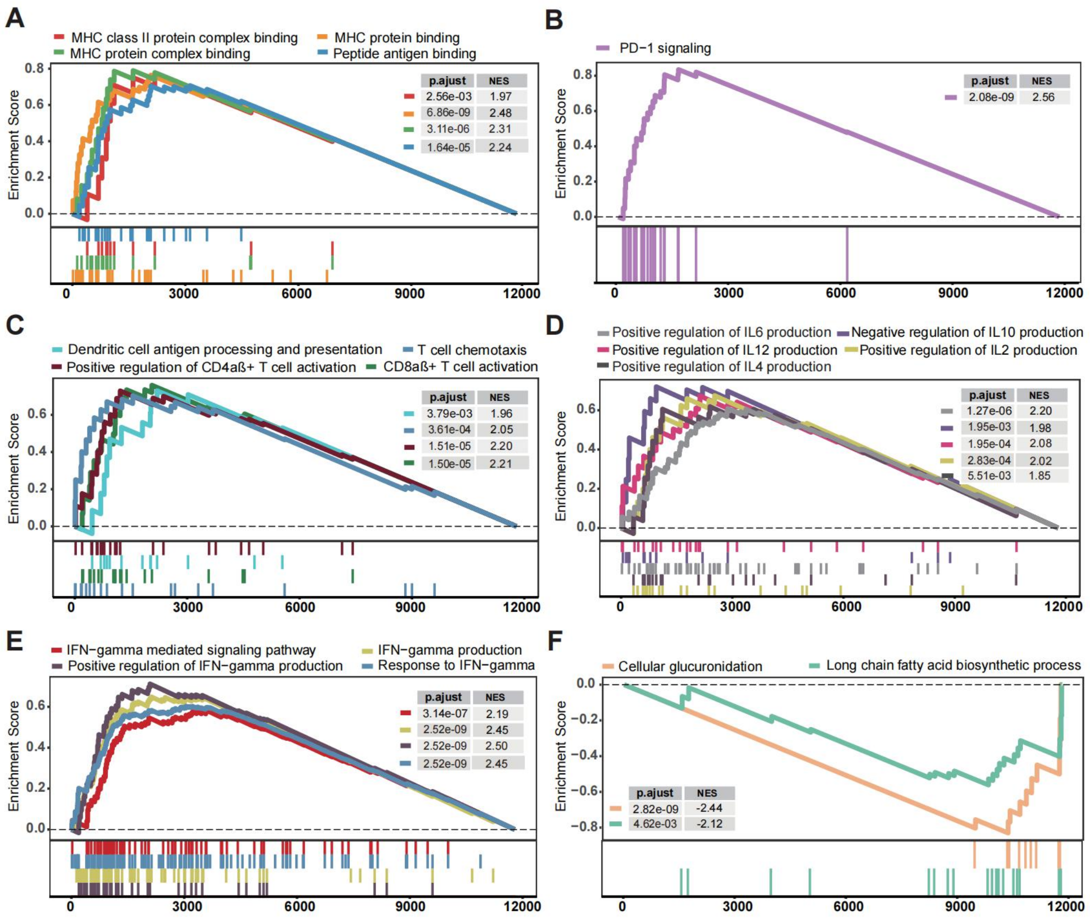

SFigure 1. Validation of transcriptome traits related to MHC-II signature in TCGA-BLCA cohort.

A-F. GSEA analysis shows the key pathways of enrichment in the MHC-H (up) and MHC-L (down) groups. Antigen processing and presentation (A), PD−1 signaling (B), immune cell activation (C), cytokine production (D) and IFN−gamma (E) related pathways were significantly upregulated in the MHC-H group, while long chain fatty acid biosynthetic process and glucuronidation (F) were significantly upregulated in the MHC-L group.

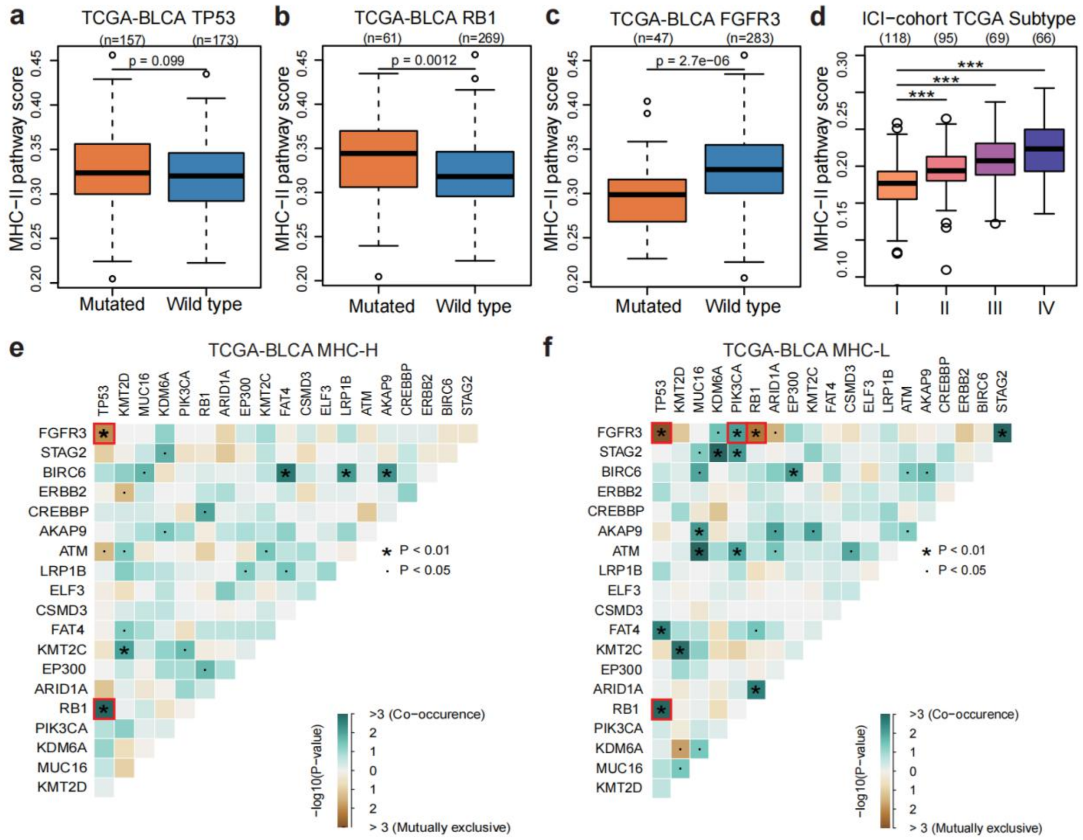

SFigure 2. Validation of genomics landscape related to MHC-II signature in the TCGA-BLCA cohort.

A-C. Boxplots showed that TP53 (A) and RB1 (B) gene mutations were correlated with high MHC-II signature in the TCGA-BLCA cohort (Mann Whitney U test, p = 0.099, p = 0.0012, respectively), while FGFR3 (C) gene mutations were significantly correlated with low MHC-II signature in the TCGA-BLCA cohort (Mann Whitney U test, p = 2.7e-06).

D. Tumor TCGA stage was significantly correlated with MHC-II signature score in the ICI-cohort. ***P < 0.001.

E-F. Concurrence (blue) and mutual exclusion (brown) between high frequency mutation genes (Top20 genes) in the MHC-H (E) and MHC-L(F) groups in the TCGA-BLCA cohort. ·p < 0.05, *p < 0.01.

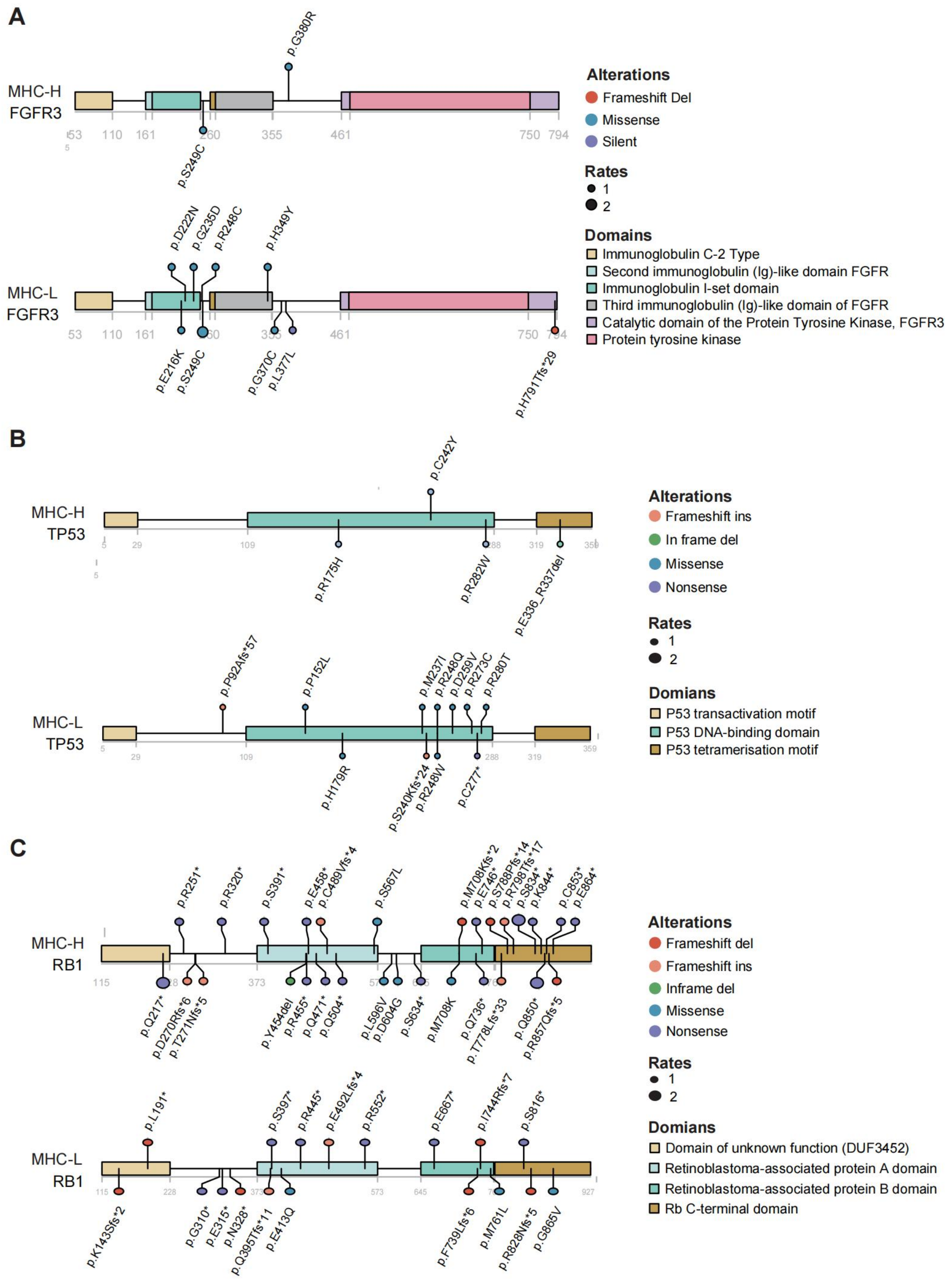

SFigure 3. The mutated loci landscape related to MHC-II signature in the TCGA-BLCA cohort.

A. Lollipop plot comparing the mutated loci of FGFR3 gene between MHC-H (upper) and MHC-L (lower) groups in the TCGA-BLCA cohort.

B. Lollipop plots comparing the mutated loci of TP53 gene between the MHC-H (upper) and MHC-L (lower) groups in the TCGA-BLCA cohort.

C. Lollipop plots comparing the mutated loci of RB1 gene between the MHC-H (upper) and MHC-L (lower) groups in the TCGA-BLCA cohort.

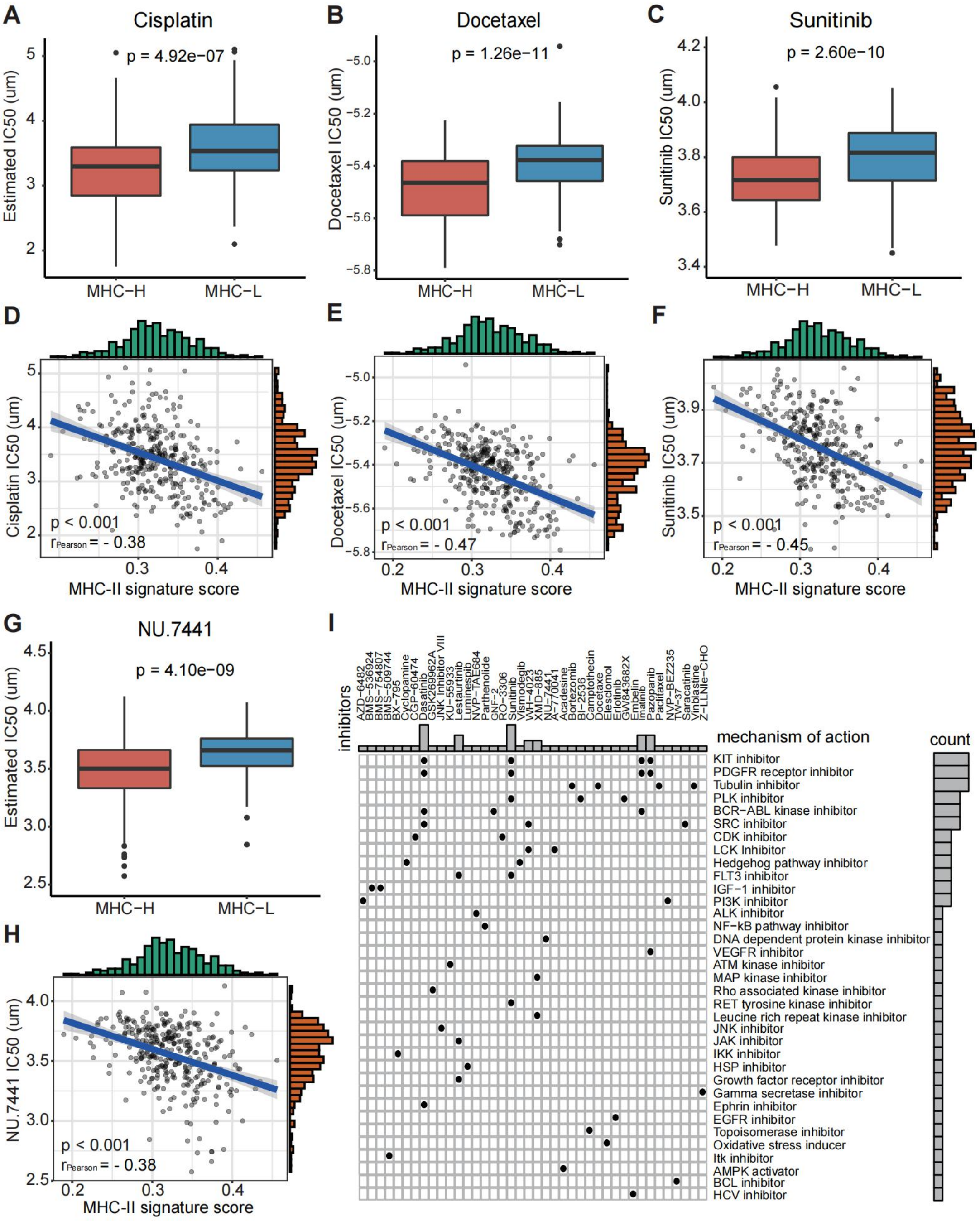

SFigure 4. Validation of the role of MHC-II signature in drug sensitivity prediction in the TCGA-BLCA cohort.

A-H. Boxplots show that the IC50 values of Cisplatin (A), Docetaxel (B), Sunitinib (C) and NU.7441 (G) were significantly lower in the MHC-H group compared to the MHC-L group (Wilcoxon.test, p = 4.92e−07, p = 1.26e−11, p = 2.60e−10, p =

4.10e−09, respectively). In addition, IC50 values of Cisplatin (D), Docetaxel (E), Sunitinib (F) and NU.7441 (H) were significantly negatively correlated with the MHC-II signature score (Pearson test, $r_{Pearson}$ = - 0.38, $r_{Pearson}$ = - 0.47, $r_{Pearson}$ = - 0.45, $r_{Pearson}$ = - 0.38, respectively, All $p < 0.001$).

I. Heatmap show the MoA (row) shared by each compound (column, n=40) in the TCGA-BLCA cohort. The MoA is sorted according to the number of compounds sharing the MoA, and a heatmap is displayed.
